# Effects of a Prosthetic Foot With Increased Coronal Adaptability on Cross-Slope Walking

**DOI:** 10.33137/cpoj.v4i1.35206

**Published:** 2021-06-25

**Authors:** B Altenburg, M Ernst, P Maciejasz, T Schmalz, F Braatz, H Gerke, M Bellmann

**Affiliations:** 1 Research Biomechanics, Ottobock SE & Co. KGaA, Göttingen, Germany.; 2 Clinical Research and Services, Ottobock SE & Co. KGaA, Duderstadt, Germany.; 3 Medical Orthobionics, Pivate University of Applied Sciences, Göttingen, Germany.; 4 Institute of Biomechanics and Orthopaedics, German Sport University Cologne, Köln, Germany.

**Keywords:** Cross-slopes, Transtibial, Amputation, Coronal Adaptation, Prosthetic Foot, Center of Pressure, External Knee Adduction Moment (EKAM)

## Abstract

**BACKGROUND:**

Walking on cross-slopes is a common but challenging task for persons with lower limb amputation. The uneven ground and the resulting functional leg length discrepancy in this situation requires adaptability of both user and prosthesis.

**OBJECTIVE(S):**

This study investigated the effects of a novel prosthetic foot that offers adaptability on cross-slope surfaces, using instrumented gait analysis and patient-reported outcomes. Moreover, the results were compared with two common prosthetic feet.

**METHODOLOGY:**

Twelve individuals with unilateral transtibial amputation and ten able-bodied control subjects participated in this randomized cross-over study. Participants walked on level ground and ±10° inclined cross-slopes at a self-selected walking speed. There were three prosthetic foot interventions: Triton Side Flex (TSF), Triton LP and Pro-Flex LP. The accommodation time for each foot was at least 4 weeks. The main outcome measures were as follows: frontal plane adaptation of shoe and prosthetic foot keel, mediolateral course of the center of pressure, ground reaction force in vertical and mediolateral direction, external knee adduction moment, gait speed, stance phase duration, step length and step width. Patient-reported outcomes assessed were the Activities specific Balanced Confidence (ABC) Scale, Prosthetic Limb Users Survey of Mobility (PLUS M) and Activities of Daily Living Questionnaire (ADL-Q).

**FINDINGS:**

The TSF prosthetic foot adapted both faster and to a greater extent to the cross-slope conditions compared to the Triton LP and Pro-Flex LP. The graphs for the mediolateral center of pressure course and mediolateral ground reaction force showed a distinct grouping for level ground and ±10° cross-slopes, similar to control subjects. In the ADL-Q, participants reported a higher level of perceived safety and comfort when using the TSF on cross-slopes. Eight out of twelve participants preferred the TSF over the reference.

**CONCLUSIONS:**

The frontal plane adaptation characteristics of the TSF prosthetic foot appear to be beneficial to the user and thus may enhance locomotion on uneven ground – specifically on cross-slopes.

## INTRODUCTION

Real-life outdoor walking of persons with a lower limb amputation is continuously challenged by uneven ground, including bumps, obstacles, slopes and cross-slopes. Cross-slopes are especially demanding,^1^ since many sidewalks are generally tilted for water drainage and often intersected with driveways. Such tilted ground induces a functional leg length discrepancy, which is an apparent problem, in particular when the prosthetic limb is positioned hillside and is effectively too long. This requires the user to perform compensatory strategies during gait.^2^ Conversely, an adaptive prosthesis may diminish compensatory user effort. In a lower limb prosthesis, the prosthetic foot is a central component which offers an individual adaptability, depending on the design and materials used.^3^

Due to their carbon structure, common energy-storing-and-returning (ESR) feet have a certain degree of flexibility, which allows for limited adaptation under load.^3^ It has been shown in different studies^4-6^ that a mechanical ankle joint can increase the range of motion in plantar and dorsiflexion and that a sophisticated microprocessor control can improve the adaptation to uneven ground.^7-9^ Most studies have focused on adaptations in the sagittal plane. Only a few studies have investigated prototype feet that adapt in the frontal plane.^10,11^ Many ESR feet are equipped with a split-toe feature that is thought to add flexibility in the frontal plane.^3^ However, it has not yet been shown that such foot design benefits individuals with lower-limb amputations walking on inclined ground. Moreover, few studies have investigated amputees’ gait on cross-slopes.^12-14^ Most recently, Villa et al. revealed compensatory strategies of lower-limb amputees during prosthetic swing when the prosthesis was positioned hillside.

Individuals with transtibial amputation (ITTAs) showed increased hip and knee flexion in the residual limb for compensation and individuals with transfemoral amputation (ITFAs) increased hip hiking and vaulting.^12^ Such vaulting strategies of ITFAs were investigated in a previous article by Villa.^13^ Starholm et al.^14^ found that ITFAs use significantly more energy when walking on a surface moderately tilted in the frontal plane compared to walking with a tilt in the sagittal plane. They assume that when the prosthesis is on the slope side it becomes functionally too long and provokes a more energy consuming gait pattern. The existing literature shows that the most-investigated situation of cross-slope walking of amputees is walking with the prosthesis hillside, whereby the focus is placed on prosthetic swing, intact side stance and required compensatory strategies.

Building on these approaches, this study focused on the biomechanics of the prosthetic stance phase in both cross-slope conditions: foot positioned hillside (provokes eversion) and foot positioned valleyside (provokes inversion). The aim was to reveal the impact of the adaptability of the prosthetic foot on kinematics and kinetics of amputee gait on cross-slopes and to analyze the effects on balance and comfort by using self-reported outcome measures. The hypothesis was that a novel foot module with high frontal plane compliance enhances the locomotion of ITTAs on uneven ground.

## METHODOLOGY

### Participants

Twelve ITTAs participated in the study. This randomized cross-over study was approved by the ethics committee of the medical faculty of the University of Göttingen, Germany. Inclusion criteria were as follows: active individuals (K-level 3, 4)^15,16^ with unilateral transtibial amputation, at least 18 months post-amputation, stable residual limb volume, stable gait pattern, aged 18 years or older. Exclusion criteria were as follows: body weight exceeding 125 kg, any conditions that severely influence performance and gait like cardiovascular diseases or present socket issues. In addition, ten able-bodied individuals were included as controls. All participants provided written informed consent.

### Prosthetic Feet

**Figure 1:**
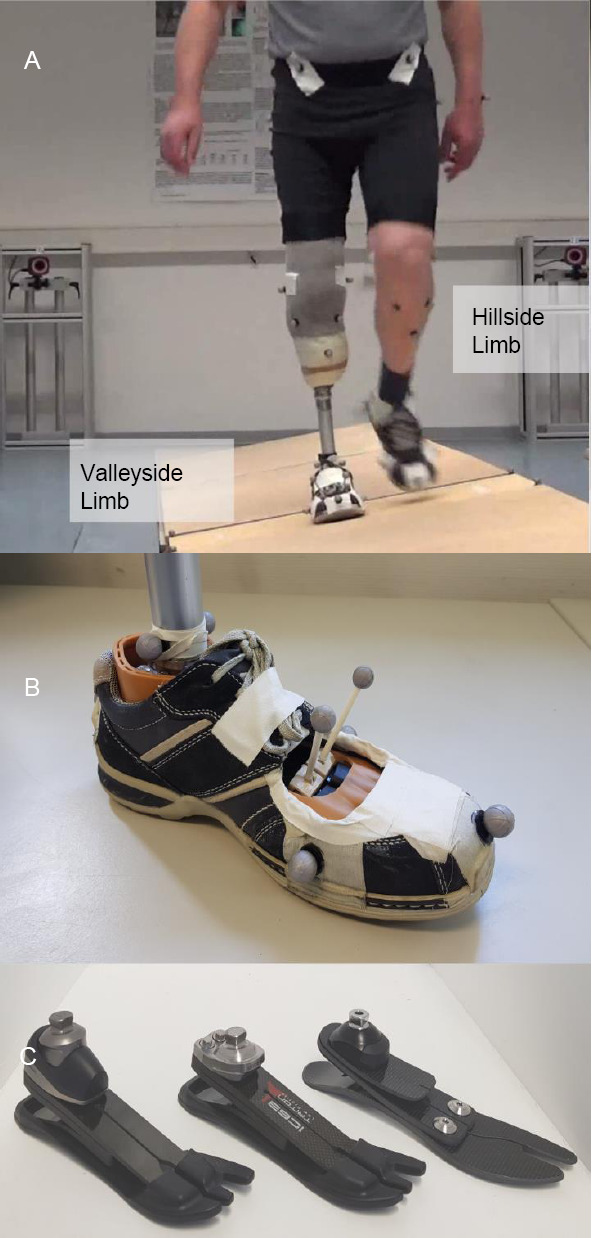
Experimental setup and prosthetic feet used. (A) shows the 8.5m cross-slope with a force plate embedded in the center of the track (shown step) and a typical participant (prosthesis is valleyside limb, intact side is hillside limb). (B) display the marker set for estimating the CoP (markers on toe and heel of shoe) and the adaptation to the tilt (shoe markers and antenna markers through a cutout in the shoe and foot shell, attached to the carbon base). (C) shows the prosthetic feet tested: Triton Side Flex (left), Triton LP (middle) and Pro-Flex LP (right).

Three ESR feet were investigated (**Figure 1**). The novel foot was the Triton Side Flex (Ottobock, Germany). The TSF features a dedicated joint for frontal plane adaptations. This joint unit enables ±10° rotation (inversion/eversion) against a progressive resistance that is provided by a torsion bar.^17^ The rotational resistance increases towards the hard stop at 10° and is not user specific. The unit is screwed on a carbon base with split toes. The overall weight (size 27, including Spectra sock and cosmetic cover) is 860 g. The build height (heel to top of the pyramid) is 109 mm. In addition, two reference feet were tested. The Triton LP (Ottobock, Germany) contains the same carbon base as the TSF with matching properties in terms of dimensions and basic flexibility. The overall weight is 690 g and the build height 86 mm. The Pro-Flex LP (Ossur, Iceland) is a direct competitor of the Triton LP in the section of low profile ESR feet for active (K3 + K4) users. It features split toes as well, which are, however, asymmetrically designed. The overall weight (660 g) and build height (90 mm) are similar to the Triton LP.

### Workflow

The study captured biomechanical data and patient-reported outcomes. Each participant tested the TSF and one reference foot. The selection of the reference foot and the order of tests (start with the TSF or reference) were randomized. All prosthetic assembling was done by the same certified prosthetist. The bench alignment followed the manufacturers’ specifications and was reproducibly done using a PROS.A. Assembly (Ottobock, Germany)^18^ followed by a static optimization^19,20^ on the L.A.S.A.R. Posture (Ottobock, Germany)^21^ and a final dynamic optimization to the prosthetist’s and participants’ satisfaction. The accommodation time for each foot was at least 4 weeks prior to performing the biomechanical assessments and patient questionnaires.

### Setup

Gait analysis measurements were gathered using a motion capture system consisting of 12 Bonita cameras (Vicon, UK, sampling rate 200 Hz) and two force plates (Kistler, Switzerland, sampling rate 1000 Hz). A dedicated marker set with 39 passive markers was applied. This enables the assessor to distinguish between the different contributions to the frontal plane adaptations of the foot including joint adaptation, carbon base deformation and shoe/foot shell deformation.^3^ For this purpose, the shoe and foot shell were modified and antenna markers were mounted directly on the carbon base (**Figure 1**). During measurements, all participants wore the same model of shoe with defined marker positions.

The studied situations were walking on level ground (using both force plates in the middle of the track) and walking on a 10° cross-slope, which was 8.5 m long and equipped with one force plate in the center. The participants were instructed to walk on the track at a self-selected, comfortable walking speed several times until eight valid recordings for each condition (level walking, four cross-slope conditions: 10° prosthesis hillside, 10° intact side hillside, 10° prosthesis valleyside, 10° intact side valleyside) were captured. The inclusion or exclusion of each recording was determined by an assessor next to the track (inclusion criteria: steady state of walking in the middle of the track, entire foot on the force plate without obviously aiming for it and without specific step length adaptation). As the physical cross-slope setup remained the same for all cross-slope measurements, the starting point was adjusted for each individual subject and both walking directions were captured. For the healthy controls both sides were measured in equal distribution in order to generate comparative data in 3 different conditions (level, hillside, valleyside).

In addition to the biomechanical measurements, participants completed a questionnaire evaluating amputees’ experience during 4 weeks or more of daily use. This questionnaire included the Activities-Specific Balance Confidence (ABC) Scale^22^ (16 questions), the Prosthetic Limb Users Survey of Mobility (PLUS-M) Scale^23,24^ (12-item short form) and a self-developed scale evaluating socket comfort and perceived safety in 40 situations of daily living (ADL-Scale). Among the situations evaluated with the ADL-Scale, there were 9 standing situations, 11 walking situations potentially affected by medial/lateral flexibility, 8 walking situations potentially unaffected by medial/lateral flexibility and 12 social activity situations. Both socket comfort and perceived safety were measured on a numerical rating scale from 0 (worst) to 10 (best). At the end of the study, participants were also asked which of the two tested feet they preferred for daily use.

### Data analysis

**Figure 2:**
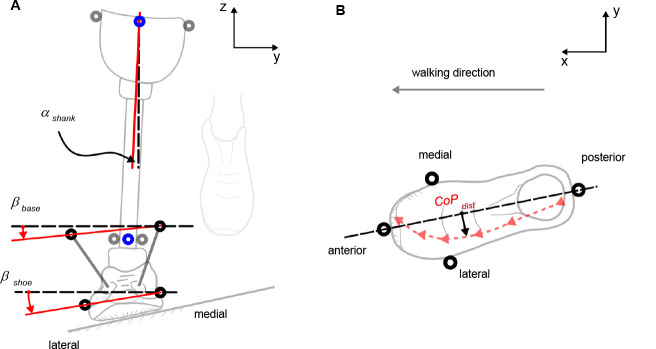
Diagram to introduce the parameters used. **(A)** Shank and foot from frontal view: Markers, symbolized by circles, were projected in the frontal plane (y-**z** plane) to calculate β_shoe_ (medial and lateral shoe marker), β_base_ (medial and lateral antenna marker) and α_shank_ (virtual center of knee and ankle marker). These parameters were used to estimate the relative adaptation α_shoe_ and α_base_ to cross-slopes. The external knee adduction moment (EKAM) was calculated using the virtual center of the knee (blue marker, middle of lateral and medial knee markers) and the ground reaction force (using cross product in lab coordinate system). **(B)** foot from transversal view (lab coordinate system): CoPdist, the orthogonal distance between the CoP course (red-arrow line) and the foot axis (black-dashed line, defined by tip and heel marker), was calculated on the cross-slope surface. Note, the cross-slope surface is tilted (10°) relative to the shown transversal plane (x-y plane) shown above.

Valid trials were further processed with VICON Nexus, customized VICON BodyBuilder (Vicon Motion System, UK) and MATLAB (R2018a, Mathwork Inc, US) scripts. The following spatiotemporal gait characteristics were considered: gait speed, step length, step width and stance phase duration. The lateral shoe markers, **Figure 2** were used to automatically calculate these parameters with VICON Nexus.^25^ Furthermore, the following kinetic parameters were determined: ground reaction force in vertical (GRFv) and mediolateral (GRFml) direction; external knee adduction moment (EKAM), considered as EKAM peak (first maximum) and EKAM impulse (EKAM integral over duration of gait cycle (GC)).

The adaptation of the shoe and carbon base in the frontal plane and the mediolateral course of the center of pressure (CoP) with respect to the foot were specific biomechanical parameters used in the study. The relative adaptation of the shoe in the frontal plane was determined in three steps. First, the angle between the projected connection line of medial and lateral shoe markers in the frontal plane and the horizontal surface was calculated (β_shoe_ in **Figure 2**). Second, the adaptation of the shoe was calculated: α'_shoe_=β_shoe_+α_shank_. Hereby α_shank_ represents the frontal shank angle (α_shank_ in Figure **2**). Third, the relative adaptation of the shoe in the frontal plane was calculated by subtracting the adaptations found for level walking from the adaptations found for cross-slope situations: α_shoe_=α'_shoe, cross-slope_-α'_shoe, level_. The calculations were performed in the laboratory coordinate system. The same calculation was carried out for the medial / lateral antenna markers, which were attached to the associated parts of the carbon base (α_base_). It was assumed that these values represent the adaptation of the carbon base of the prosthetic foot.^3^

The course of the CoP was estimated, first, by projecting the CoP of the force plate and the foot axis (defined by tip and heel marker on the shoe) to the surface and, second, by calculating the orthogonal distance of the projected CoP to the projected marker line (CoP_dist_ in **Figure 2**).

### Statistics

For each situation, individual means (calculated from single trials) and group means (calculated from individual means) of the analyzed parameters were determined. To compare the effects of the different prosthetic foot types (reference feet vs. TSF) for certain setups (level, 10° hillside, 10° valleyside) a paired T-test was performed. If the assumed normal distribution (Shapiro-Wilks test) was not given, a non-parametric Wilcoxon test was performed. To account for multiple testing, the alpha level was set to 1%. The statistical analysis was performed with IBM SPSS Statistics (IBM Corp., US). We did not conduct a statistical analysis of the effects of the cross-slopes angle within the groups, i.e. level vs 10° hillside vs 10° valleyside, nor did we consider a statistical comparison between ITTAs and control subjects. We did, however, perform a statistical analysis of the ABC, PLUS-M and ADL-Scale scores. The ADL scores were separately determined for each activity block (standing, walking – non-m/l, walking – m/l, and social activities) and each dimension (safety, comfort) – resulting in eight ADL scores per person and tested foot. The individual scores of participants obtained with different feet were compared using the paired T-test.

## RESULTS

Twelve ITTAs with activity level K3 (Medicare Functional Classification Level) or higher, participated in this study. Further detailed demographic data are shown in **Table 1**. In addition 10 control subjects (age: 29±7 years; weight: 83±15 kg; height: 183±11 cm) were also recruited for the study.

**Table 1:** Demographic data of study participants with transtibial amputation and allocated prosthetic feet (randomized process)

Participant	Age	Weight (kg)	Height (m)	Gender	K-Level	Socket suspension	Foot #1	Foot #2
S01	51	69	1,83	m	3	Suction, One way valve	TSF	Triton LP
S02	61	125	1,80	m	3	Soft socket, supracondylar	TSF	Pro-Flex LP
S03	38	88	1,68	m	4	Suction, one way valve	Pro-Flex LP	TSF
S04	77	86	1,75	m	3	Pin lock	TSF	Pro-Flex LP
S05	47	50	1,56	w	3	Suction, one way valve	Pro-Flex LP	TSF
S06	59	86	1,78	m	3	Suction, one way valve	TSF	Pro-Flex LP
S07	44	68	1,68	w	3	Suction, one way valve	Triton LP	TSF
S08	52	78	1,77	m	3	Suction, one way valve	TSF	Triton LP
S09	37	90	1,84	m	3	Suction, one way valve	Triton LP	TSF
S10	64	99	1,88	m	3	Suction, one way valve	Triton LP	TSF
S11	57	79	1,80	m	4	Suction, one way valve	Pro-Flex LP	TSF
S12	47	97	1,83	m	4	Pin lock	TSF	Triton LP
**Mean**	52,8	83,9	1,77					
**SD**	11,5	19,4	0,09					
**Range**	37-77	50-125	1,56-1,88					

The spatiotemporal parameters provided in **Table 2** showed no statistically significant differences between the TSF and reference feet for any condition. Gait speed and step width tended to decrease from level to slope conditions for all feet. Control subjects walked on average with higher gait speed, step length, and step width in all conditions (not statistically analyzed).

**Table 2:** Spatiotemporal parameters of the TSF and reference feet for level walking and 10° cross-slope conditions. No statistically significant differences between foot types were found (p<0.01)

Prosthetic foot	Condition	Gait velocity (m/s)	Step length (m)	Stance phase duration (% GC)	Step width (m)
**TSF**	Level	1.27 ± 0.12	0.74 ± 0.05	60.7 ± 1.3	0.22 ± 0.03
	10° hillside	1.25 ± 0.14	0.75 ± 0.05	61.1 ± 1.2	0.21 ± 0.03
	10° valleyside	1.23 ± 0.13	0.71 ± 0.05	60.3 ± 1.3	0.20 ± 0.02
**Reference**	Level	1.27 ± 0.14	0.73 ± 0.06	61.3 ± 0.9	0.22 ± 0.02
	10° hillside	1.24 ±0.14	0.75 ± 0.06	61.1 ± 1.4	0.21 ± 0.02
	10° valleyside	1.25 ± 0.15	0.72 ± 0.07	60.7 ± 1.2	0.20 ± 0.03
**Controls**	Level	1.44 ± 0.15	0.79 ± 0.09	61.7 ± 1.1	0.27 ± 0.03
	10° hillside	1.42 ± 0.19	0.79 ± 0.08	61.8 ± 1.4	0.25 ± 0.04
	10° valleyside	1.43 ± 0.19	0.77 ± 0.08	60.8 ± 1.4	0.26 ± 0.03

**Figure 3:**
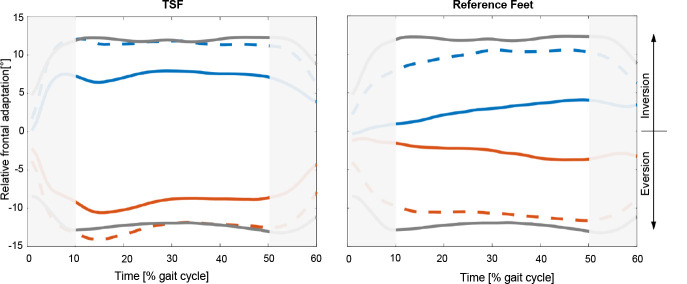
Relative frontal adaptation on the cross-slope for Triton Side Flex (left) and reference feet (right) for 10% to 50% GC (main loading interval). The solid red (hillside) and solid blue (valleyside) curves show the adaptation of the prosthesis via the antenna markers (α_base_). The grey (control subjects) and dashed (prosthetic side) curves show the adaptation determined by the markers on the shoes (β_shoe_).

The frontal plane adaptations of the studied feet and shoes in cross-slope conditions during stance are represented in **Figure 3**. For the TSF, a major and continuous adaptation from the beginning of mid stance was found, whereas the reference feet showed only minor adaptations after loading response, constantly increasing with progressing stance.

The amount of measured adaptation at mid stance (30% GC) was significantly (p<0.01) higher with the TSF compared to the reference feet in all slope conditions (**Table 3**). The measured shoe adaptation showed similar characteristics with a smaller difference between the studied feet. The TSF reached the same amount of adaptation as the controls.

**Table 3:** Parameters of the prosthetic side for the different setups at the first maximum peak (GRFv, EKAM peak), whole GC (EKAM impulse) and at 30% GC (GRFml, α_base_, α_shoe_, CoP_dist_). Statistically significant differences between foot types are marked bold (p<0.01) or bold and * (p<0.001)

Parameter	GRFv (% bw)	GRFml (%bw)	α_base_(deg)	α_shoe_(deg)	CoP_dist_(mm)	EKAM peak	EKAM impulse
Level TSF	108 ± 11	4.4 ± 1.3	-	-	**9 ± 5**	0.34 ± 0.12	0.09 ± 0.04
Level reference feet	108 ± 7	4.5 ± 1.2	-	-	**14 ± 7**	0.32 ± 0.11	0.09 ± 0.04
Level controls	111 ± 5	2.0 ± 0.8	-	-	9 ± 6	0.49 ± 0.10	0.19 ± 0.05
Hillside TSF	108 ± 13	4.2 ± 1.3	-8.8 ± 1.6 ^*^	**-11.9 ± 0.8**	13 ± 5 ^*^	0.27 ± 0.13	0.08 ± 0.04
Hillside reference feet	109 ± 12	4.5 ± 1.1	-2.1 ± 0.6 ^*^	**-10.7 ± 0.8**	24 ± 5 ^*^	0.26 ± 0.13	0.08 ± 0.04
Hillside controls	107 ± 6	2.2 ± 0.8	-	-12.0 ± 0.9	10 ± 7	0.38 ± 0.10	0.16 ± 0.06
Valleyside TSF	112 ± 12	**3.4 ± 1.5**	7.9 ± 1.8 ^*^	**11.9 ± 2.0**	13 ± 6 ^*^	0.38 ± 0.14	**0.11 ± 0.03**
Valleyside reference feet	112 ± 11	**2.4 ± 1.2**	2.9 ± 1.0 ^*^	10.6 ± 1.2	2 ± 5 ^*^	0.40 ± 0.12	**0.14 ± 0.03**
Valleyside controls	112 ± 7	1.2 ± 0.7	-	12.0 ± 1.0	5 ± 6	0.56 ± 0.16	0.22 ± 0.06

**Figure 4:**
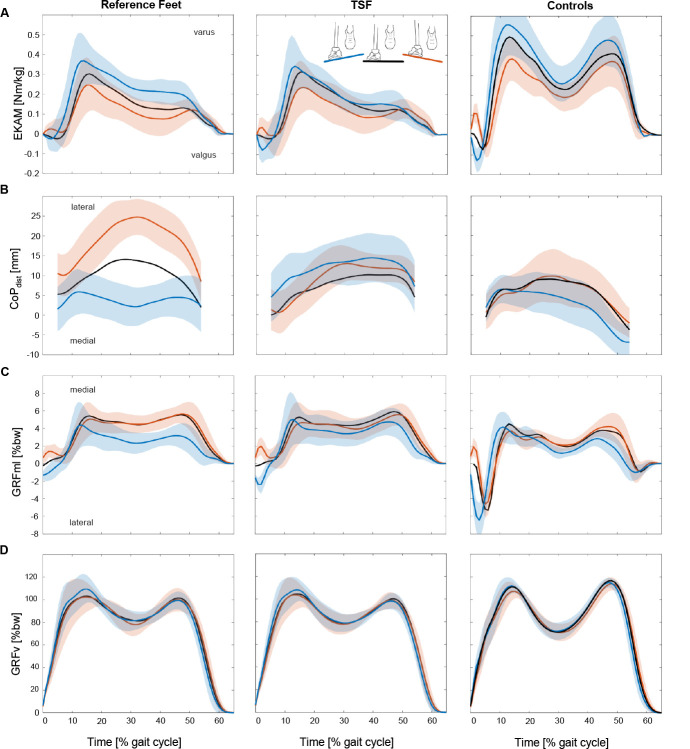
Parameters for the different studied situations. **(A)** EKAM, **(B)** mediolateral CoP course, **(C)** mediolateral ground reaction forces and **(D)** vertical ground reaction forces. The left column shows the prosthetic side values for the reference feet, the middle column the prosthetic side values for the TSF foot and the right column show the control subjects. The blue curves indicate the valleyside situations, the red curves indicate the hillside situations and the black curves indicate level walking. The associated standard deviations are given as shared areas for the blue and red curves.

The EKAM impulse was significantly reduced for the valleyside condition with the TSF. No differences were found for hillside and level walking (**Table 3**, **Figure 4**). EKAM peaks showed no differences in all conditions. Control subjects presented notably higher values of peak EKAM in all conditions.

The CoP paths of the control subjects showed a medial shift when the foot in question was positioned valleyside, but a close grouping for the hillside position and level walking. The TSF produced a similar grouping for level and hillside conditions, but a lateral shift for the valleyside condition. ESR feet revealed a different CoP path pattern with a distinct laterally shifted curve for the hillside and a medially shifted curve for the valleyside condition. A comparison of ESR and TSF feet demonstrated significantly different CoP paths for all conditions (**Table 3**).

GRFv showed no differences between the feet in all conditions. There was a trend towards an increased first maximum for the valleyside condition. GRFml differed significantly between the investigated feet in the valleyside condition (30% GC).

The results of the patient-reported outcomes are shown in **Table 4**. They found significantly (p<0.05) higher ratings for the TSF foot for perceived comfort while standing and perceived comfort and safety while walking in situations potentially affected by medial/lateral flexibility (ADL Scale). All other ADL sub-scales, as well as ABC and PLUS-M, tended towards higher ratings when using the TSF foot, but did not attain statistical significance. As far as foot preference was concerned, eight participants preferred the TSF, three participants preferred one of the reference feet (1x Triton LP, 2x Pro-Flex LP), and one participant had no preference.

**Table 4:** Summary of the scores in the patient-reported outcomes for reference feet and the TSF. Significant differences in a parameter are marked bold for the p values (p<0.05).

Analyzed parameter:	n	Reference feet	TSF (mean ± SD)	p
**ABC (Balance confidence)**	12	86.6 ± 8.6	89.2 ± 8.4	0.14
**PLUS-M (Mobility)**	12	56.6 ± 7.3	57.3 ± 7.8	0.67
**ADL: Standing situations (9 questions)**				
Perceived comfort	12	8.4 ± 1.0	9.1 ± 0.8	0.02
Perceived safety	12	8.8 ± 0.8	9.2 ± 0.7	0.11
**ADL: Walking in situations potentially affected by the medial/lateral flexibility (11 questions)**				
Perceived comfort	12	8.3 ± 1.1	8.9 ± 0.8	0.02
Perceived safety	12	8.4 ± 1.1	9.0 ± 0.9	0.04
**ADL: Walking in situations potentially not affected by the medial/lateral flexibility (8 questions)**				
Perceived comfort	12	8.3 ± 0.9	8.8 ± 0.8	0.11
Perceived safety	12	8.4 ± 1.0	8.7 ± 1.0	0.34
**ADL: Social activities (12 questions)**				
Perceived comfort	12	8.3 ± 1.0	8.9 ± 0.8	0.05
Perceived safety	12	8.6 ± 0.9	8.9 ± 0.9	0.12

## DISCUSSION

The aim of this study was to investigate the effects of a novel prosthetic foot, with adaptability on cross-slope surfaces, using instrumented gait analysis and patient-reported outcomes.

Several results support the initial hypothesis that a foot module with easily accessible frontal plane adaptation can enhance locomotion on uneven ground. First, the early and extensive adaptation at the beginning of stance found in this study appears to be of importance and agrees with the findings of Yeates^10^ who suggested an improved balance on uneven ground derived from greater frontal adaptation in early stance. Here the TSF adapts both earlier (at loading response, 10% GC) and more extensively and keeps this adaptation until the end of stance (**Figure 3**). The users felt directly a different foot behavior when stepping on the cross-slope and reported a higher level of perceived safety. With the common ESR split toe concept of the measured reference feet, the maximum adaptation occurs at the end of stance with maximum toe load. The absolute value of adaptation is not exactly known since the measured position of the antenna markers fixed on the carbon base always reflect a combined adaptation of rotation of the carbon base, toe shift and toe twist.^3^ Nevertheless, the timing of adaptation is not affected. The frontal plane adaptation of the shoes reveals similar characteristics as the carbon bases with constant values for the TSF, but increasing adaptation as stance progresses for standard ESR feet.

However, the difference in adaptation is smaller compared to the carbon base data. This suggests movement, like tilt between carbon base, foot cover and shoe, that is usually not detected with conventional marker placement on the shoes. Consequently, every shoe as well as every prosthetic foot cover contributes to the overall adaptation. The data for shoe adaptation shows an overshoot in value for the TSF and control subjects (12° value on a 10° cross-slope). Load-dependent shoe-sole compression and a different shank orientation (by leaning to a side) when walking on cross-slopes are plausible reasons for this effect.

The EKAM is a clinically relevant parameter, since its first peak has been positively associated with medial compartment knee osteoarthritis (OA).^26-29^ The EKAM impulse is also commonly studied in conjunction with OA^29-32^ According to Chang et al.^33^, it might even be the more comprehensive indicator of cumulative medial compartment loading during gait. This study showed a significantly reduced EKAM impulse for the valleyside condition using the TSF, but no differences in EKAM peak (**Table 3**, **Figure 4A**). However, compared to the clearly higher moments generated by the control subjects, the overall impact on OA risk appears negligible. Still, this effect of prosthetic side knee load reduction for valleyside conditions might increase in similar everyday life loading scenarios with higher gait speed or varying step width.^34^ In general, the clearly higher gait speed and wider step width of the control subjects has to be considered when comparing absolute knee loading.

The CoP path shows clear foot-dependent differences (**Figure 4B**). It can be assumed that the additional joint of the TSF quickly adapts, similar to the subtalar joint of the control subjects, causing similarly grouped CoP paths for the TSF and control subjects. The limited adaptation capability of the low profile ESR feet forces a main load transfer towards the lateral rim of the foot base (hillside condition) or towards the medial rim (valleyside condition), respectively, causing clearly different CoP paths compared to the level walking condition. The authors assume that these deviations require more compensatory strategies by the user to safely walking on cross-slopes. The GRFml also showed a close grouping for the TSF and control subjects (**Figure 4C**). This effect might facilitate more predictable steps with less control effort on cross-sloped surfaces or similar terrain.

For the hillside conditions, there were no foot-dependent differences found for EKAM and GRFml, despite clear differences in frontal plane adaptation, COP paths and reported advantages while using the TSF. It can be assumed that the functional leg length discrepancy (35 mm longer prosthetic side on 10° cross-slope, 20 cm step width) is a major problem to overcome for the user that presumably requires higher control and energetic efforts. According to Walsh et al.,^2^ leg length discrepancies greater than 5 mm require compensatory strategies during gait. The additional joint in the TSF allows for an effective shortening of about 5 mm in this condition which is a small proportion of the estimated 35 mm leg length discrepancy but seems to positively impact perceived comfort and safety. Still, it did not translate into the measured parameters EKAM and GRFml.

The patient-reported outcomes favored the TSF. However, it is noticeable that all ratings were found to be close to the maximum scores of the scales with relatively small differences between the feet tested. It has to be considered that all participants were active ambulators with highly functional components in their current prostheses and did not report major limitations in their everyday lives. They were all considered high-functioning, safe walkers. Nevertheless, statistically significant differences were found in 3 scales. It is believed that the increased perceived safety of the TSF is based on the fast adaptation at low loading and the resulting consistent GRFml (**Figure 4C**) on sloped surfaces. The reduced control effort may lead to diminished movement in the residual limb-socket interface and could be the reason for the increased perceived comfort. The preference question at the end of the study revealed, on the one hand, a clear preference by 8 of the 12 participants for the TSF. On the other hand, interesting arguments for its rejection were offered by the other participants. Two participants preferred the Pro-Flex LP because it was perceived to provide a more comfortable rollover. One participant preferred the Triton LP since he had an unstable knee and could not stabilize it in the frontal plane when using the TSF. This may hint at a possible contraindication for fitting such adaptive foot components that warrants further study.

### Study limitations

Due to the long accommodation time (at least 4 weeks) for the test prosthesis, a blinding of the foot condition was not practicable, which introduced a potential bias in favor of the novel foot (expectation). The use of two different reference feet and grouping them into one reference is a limitation in methodology since it is to be assumed that both reference feet do not perform identically. The healthy control population was not an exact match to the experimental population in terms of age. This may impact gait characteristics.

## CONCLUSION

This is the first study analyzing prosthetic side loading during cross-slope walking of individuals with transtibial amputation which has provided new quantitative results. Although the obvious problem of a functional leg length discrepancy during cross-slope walking cannot entirely be solved by the novel foot studied, the results suggest an improved frontal plane adaptability of the prosthetic foot. In particular a comprehensive adaptation starting at low loading in early stance may enhance locomotion on cross-slopes such as uneven ground.

## DECLARATION OF CONFLICTING INTERESTS

Mr. Björn Altenburg, Dr. Michael Ernst, Dr. Pawel Maciejasz, Dr. Thomas Schmalz and Dr. Malte Bellmann are full time employees of Ottobock SE & Co. KGaA.

## AUTHOR CONTRIBUTION

- **Björn Altenburg:** study design, prosthetic fittings, gait lab measurements, data interpretation, draft manuscript.
- **Michael Ernst:** gait lab measurements, biomechanical data analysis, data interpretation, draft manuscript.
- **Pawel Maciejasz:** preparation, evaluation and analysis of the outcome measurements.
- **Thomas Schmalz:** supervision, revise manuscript.
- **Frank Braatz:** supervision, revise manuscript.
- **Henrik Gerke:** gait lab measurements, biomechanical data analysis.
- **Malte Bellmann:** study design, supervision, data interpretation, revise manuscript.

## SOURCES OF SUPPORT

None.

## ETHICAL APPROVAL

This randomized cross-over study was approved by the Ethics Committee of the Medical Faculty of the University of Göttingen, Germany. All participants provided written informed consent.
